# Continuity of GP care for patients with dementia: impact on prescribing and the health of patients

**DOI:** 10.3399/BJGP.2021.0413

**Published:** 2022-01-25

**Authors:** João Delgado, Philip H Evans, Denis Pereira Gray, Kate Sidaway-Lee, Louise Allan, Linda Clare, Clive Ballard, Jane Masoli, Jose M Valderas, David Melzer

**Affiliations:** Epidemiology and Public Health, College of Medicine and Health;; College of Medicine and Health, University of Exeter and St Leonard’s Research Practice, Exeter.; St Leonard’s Research Practice, Exeter.; St Leonard’s Research Practice, Exeter.; Centre for Research in Ageing and Cognitive Health, College of Medicine and Health.; Centre for Research in Ageing and Cognitive Health, College of Medicine and Health.; College of Medicine and Health;; Epidemiology and Public Health, College of Medicine and Health;; Health Services & Policy Research Group, College of Medicine and Health;; Epidemiology and Public Health, College of Medicine and Health, University of Exeter, Exeter.

**Keywords:** comorbidity, continuity of patient care, delirium, dementia, general practice, prescribing

## Abstract

**Background:**

Higher continuity of GP care (CGPC), that is, consulting the same doctor consistently, can improve doctor–patient relationships and increase quality of care; however, its effects on patients with dementia are mostly unknown.

**Aim:**

To estimate the associations between CGPC and potentially inappropriate prescribing (PIP), and with the incidence of adverse health outcomes (AHOs) in patients with dementia.

**Design and setting:**

A retrospective cohort study with 1 year of follow-up anonymised medical records from 9324 patients with dementia, aged ≥65 years living in England in 2016.

**Method:**

CGPC measures include the Usual Provider of Care (UPC), Bice–Boxerman Continuity of Care (BB), and Sequential Continuity (SECON) indices. Regression models estimated associations with PIPs and survival analysis with incidence of AHOs during the follow-up adjusted for age, sex, deprivation level, 14 comorbidities, and frailty.

**Results:**

The highest quartile (HQ) of UPC (highest continuity) had 34.8% less risk of delirium (odds ratio [OR] 0.65, 95% confidence interval [CI] = 0.51 to 0.84), 57.9% less risk of incontinence (OR 0.42, 95% CI = 0.31 to 0.58), and 9.7% less risk of emergency admissions to hospital (OR 0.90, 95% CI = 0.82 to 0.99) compared with the lowest quartile. Polypharmacy and PIP were identified in 81.6% (*n* = 7612) and 75.4% (*n* = 7027) of patients, respectively. The HQ had fewer prescribed medications (HQ: mean 8.5, lowest quartile (LQ): mean 9.7, *P*<0.01) and had fewer PIPs (HQ: mean 2.1, LQ: mean 2.5, *P*<0.01), including fewer loop diuretics in patients with incontinence, drugs that can cause constipation, and benzodiazepines with high fall risk. The BB and SECON measures produced similar findings.

**Conclusion:**

Higher CGPC for patients with dementia was associated with safer prescribing and lower rates of major adverse events. Increasing continuity of care for patients with dementia may help improve treatment and outcomes.

## INTRODUCTION

Dementia affects 2–3% of 65-year-olds, and 30–50% of people aged ≥85 years.^1^^,^^2^ Patients diagnosed with dementia often have additional health conditions (comorbidity) that complicate treatment plans, placing them at higher risk of polypharmacy and potentially inappropriate prescribing (PIP),^3^^,^^4^ and are more dependent on healthcare services.^5^

Continuity of GP care (CGPC) refers to care over time by the same GP. Continuity of care fosters a good working relationship between patient and doctor, and a sense of responsibility, especially if the GP is the named and accountable GP.^6^^,^^7^ Lower continuity of care is associated with poorer medication management^8^ and worse health outcomes, including increased mortality.^9^^,^^10^ Improving care for patients with dementia is regarded as a priority for healthcare delivery.^11^ However, limited evidence is available on the relationship between CGPC, treatment, and health outcomes in dementia.

Clinical Practice Research Datalink (CPRD) data were analysed (a large dataset of patient records from general practice) to investigate the impact of CGPC on treatment and health outcomes in patients with dementia. Associations between CGPC and the incidence of AHOs were estimated, and the effect of CGPC on the management of comorbid conditions (including polypharmacy and PIP) was explored.

## METHOD

This was a retrospective cohort study using anonymised medical records from patients in general practice available in CPRD living in England. Records encompass symptoms, diagnoses, and prescribed drugs. Personal identifiers for health care allow for identifying consultations with a specific GP. The CPRD is broadly representative of England’s older population.^12^^,^^13^ CPRD data linked to NHS Hospital Episode Statistics (HES) admission data, the UK government Office for National Statistics death certificate register, and quintiles of English Index of Multiple Deprivation based on individual postcode were used.

### Population

Individuals diagnosed with dementia at any time before the study start date (1 January 2016), (Supplementary Figure S1) were included. Diagnosis from primary or secondary care were accepted (diagnosis codes in Supplementary Tables S1 and S2). At the study start date, all patients were aged ≥65 years, registered with a practice, and had at least three consultations (required for calculating continuity) during a 1-year lead-in period (1 January 2015 until 31 December 2015).

Individuals with young-onset dementia or rare forms of dementia including Creutzfeldt–Jakob disease, frontotemporal dementia, and Huntington’s disease were excluded as these are distinct presentations of dementia.^14^^,^^15^ Participants were followed for a maximum of 1 year from study start date up to 31 December 2016.

**Table:** How this fits in

Evidence is limited about the potential positive effects of higher continuity of general practice care (CGPC) in patients with dementia. There is no cure for dementia, so finding elements of care that make a difference to patients remains a priority. Patients with dementia in the highest CGPC quartile were 34.8% less likely to develop delirium, 57.9% less likely to develop incontinence, and 9.7% less likely to have an emergency admission to hospital, compared with the lowest quartile. Higher CGPC was also associated with lower medication burden and fewer potential inappropriate prescriptions. This study produced evidence that higher continuity of care may contribute to improved clinical management, and to the health and quality of life of patients with dementia.

### Continuity of GP care (CGPC)

CGPC was measured in the lead-in period, 1 year before the study start date. It focused on GP consultations, disregarding other providers (for example, nurses). CGPC was estimated using:

- the Usual Provider of Care (UPC) Index, the proportion of a patient’s contacts with their most frequently seen GP;^16^^,^^17^
- the Bice–Boxerman Continuity of Care (BB) Index, the dispersion of consultations among GPs;^16^^,^^17^ and
- the Sequential Continuity of Care (SECON) Index, the proportion of sequential consultations with the same GP, that is, the same doctor providing the previous and current consultation.^16^^,^^17^

Indices produce a score between zero (no continuity) to one (perfect continuity).

### Comorbidities and frailty

In total, 14 comorbidities that are covered in the NHS Quality and Outcomes Framework, a programme to improve the quality of GP recording,^18^ were included (Table 1). Diagnoses were accepted at any time before the study start date in either primary or secondary care (for cancer, only records from 5 years before the study start date were considered since cancer survivors without recurrence of disease after 5 years were considered to be without cancer). Frailty was measured by the Electronic Frailty Index, an algorithm that uses GP records to classify patients as fit (non-frail), mild, moderate, or severe frailty, based on the accumulation of 36 deficits.^19^

**Table 1.** Baseline characteristics of those with a diagnosis of dementia

**Characteristic**	**Patients with dementia**
*n*	9324

Age, years, mean (SD)	84.5 (7.4)

Female, *n* (%)	6124 (65.7)

Quintiles of Index of Multiple Deprivation, *n* (%)	
1 (Most deprived)	2053 (22.0)
2	1960 (21.0)
3	2209 (23.7)
4	1842 (19.8)
5 (Least deprived)	1258 (13.5)
Missing	2 (0.0)

At least one GP consultation in nursing home, *n* (%)	821 (8.8)

Chronic conditions, *n* (%)	
Atrial fibrillation	2087 (22.4)
Asthma	1298 (13.9)
Cancer (5 years)	706 (7.6)
Chronic kidney disease	2860 (30.7)
Chronic obstructive pulmonary disease	1150 (12.3)
Coronary heart disease	2642 (28.3)
Depression	3006 (32.2)
Diabetes mellitus type 2	1924 (20.6)
Epilepsy	377 (4.0)
Heart failure	1271 (13.6)
Hypertension	6570 (70.5)
Hypothyroidism	1409 (15.1)
Severe mental illness	489 (5.2)
Stroke	2167 (23.2)

Number of comorbidities, *n* (%)	
0	735 (7.9)
1	1489 (16.0)
2	1911 (20.5)
≥3	5189 (55.7)

Electronic Frailty Index, *n* (%)	
Fit	2241 (24.0)
Mild	4881 (52.3)
Moderate	1891 (20.3)
Severe	311 (3.3)

Polypharmacy, *n* (%)	7612 (81.6)

Extreme polypharmacy, *n* (%)	3949 (42.4)

Potentially inappropriate prescribing, *n* (%)	7027 (75.4)

Incidence of adverse health outcomes, *n* (%)	
Death	1827 (19.6)
Emergency admission to hospital	3644 (39.1)
Delirium	488 (5.2)
Anaemia	192 (2.1)
Falls	720 (7.7)
Fragility fracture	253 (2.7)
Incontinence	329 (3.5)
Osteoarthritis	122 (1.3)
Osteoporosis	93 (1.0)
Pneumonia	716 (7.7)

*SD = standard deviation.*

### Management of comorbid conditions

Number of prescriptions and PIP were characterised during the lead-in period. Prescriptions in the 3 months before the study start date were counted, based on chapters 1–15 of the British National Formulary, excluding repeat prescriptions.^20^ Polypharmacy and extreme polypharmacy are defined as ≥5 and ≥10 prescriptions, respectively. PIP was defined as the prescription of any combination of drugs deemed potentially harmful by STOPP/START version 2 criteria. In total, 56 of the 80 defined criteria were implemented using methods described by Delgado *et al.*^4^

### Adverse health outcomes (AHOs)

A list of AHOs was selected that are common in older patients, which were previously used to estimate the health impact of PIP in people living with dementia.^4^ Incidence of AHOs was recorded during 1 year of follow-up; the selected list included all-cause mortality, emergency admissions to hospital and diagnoses of delirium, anaemia, falls, fractures, incontinence, osteoarthritis, osteoporosis, and pneumonia recorded in primary care records. AHOs were recorded at first occurrence during the follow-up period.

### Statistical analysis

Quartiles of CGPC measures (highest quartile [HQ], high intermediate quartile [HIQ], low intermediate quartile [LIQ], and lowest quartile [LQ]) were used to characterise levels of continuity.

Analyses on associations between CGPC and baseline data (for example, demographics, prescriptions data, and PIP) used two-sided Student’s *t*-test and linear regression models for continuous variables, Wilcoxon rank-sum test and negative binomial models for count data (for example, count of prescriptions and PIP), and χ^2^ and logistic regression models for categorical variables. Survival analyses tested associations between CGPC and incidence of AHOs during follow-up. Cox proportional hazards models were used for mortality, and Fine and Gray competing risk models, with mortality as competing risk, for all other longitudinal outcomes.^21^

All models were adjusted for age (squared), sex, quintiles of multiple deprivation, diagnosis of 14 chronic conditions (listed in Table 1), frailty classification based on the Electronic Frailty Index, and the number of consultations during the lead-in period (a proxy of medical needs). Survival analyses were also adjusted for the prior presence of the target outcome when analysing repeatable events.

Sensitivity analyses (Supplementary Tables S3–S5) include:

- restricting analyses to individuals living in the community, defined as individuals without a recorded GP consultation in a nursing or residential home during the lead-in period;
- excluding the first 6 months of follow-up (to test for reverse causation); and
- exclusion of the first and fifth quintile of number of consultations in the lead-in period.

Statistical significance was set at a *P*-value <0.05. All analyses were conducted using Stata Version 15.

## RESULTS

There were 9324 individuals who were diagnosed with dementia before the study start date (age mean 84.5 years, SD 7.4, 65.7% female) and met the inclusion criteria (Table 1). Patients with dementia had an average of 14.5 (SD 9.9) consultations with a GP during the lead period (data not shown). In total, 92.1% (*n* = 8589) had at least one additional comorbidity to dementia, with 55.7% having three or more additional conditions. Participants were followed on average for 327.2 days, with 80.4% (*n* = 7497) of participants followed for the maximum full calendar year (data not shown). Polypharmacy and PIP were identified in 81.6% (*n* = 7612) and 75.4% (*n* = 7027) of the sample, respectively. As shown in Table 1, 8.8% (*n* = 821) were nursing or residential home residents.

### Continuity of GP care (CGPC) and adverse health outcomes (AHOs)

During follow-up, 1827 of patients (19.6%) died (Table 1). The most commonly recorded AHO during follow-up was emergency admission to hospital (*n* = 3644, 39.1%), followed by falls (*n* = 720, 7.7%), pneumonia (*n* = 716, 7.7%), delirium (*n* = 488, 5.2%), and incontinence (*n* = 329, 3.5%). The least recorded was osteoporosis (*n* = 93, 1.0%).

**Figure 1.**
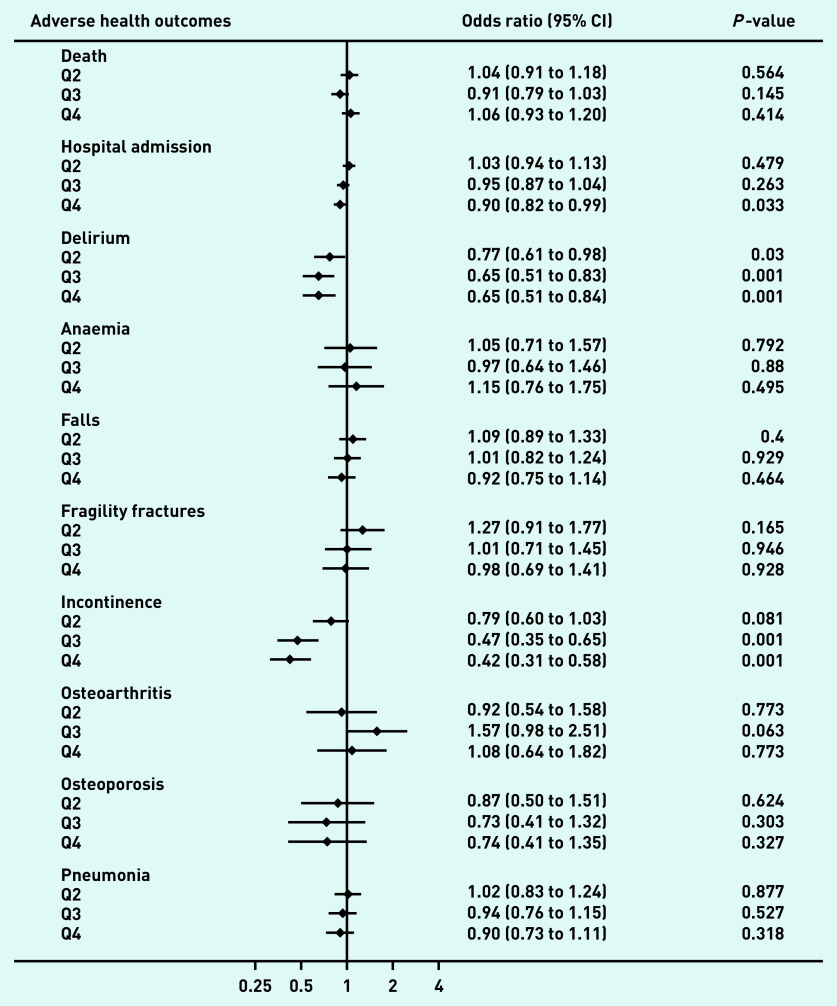
***Relative risk of incidence of adverse health outcomes by quartile of Usual Provider of Care Index (reference: quartile with least continuity) in patients with dementia.*** ^a^ *^a^* ***All analyses were adjusted for age, sex, the diagnosis of 14 chronic conditions and prior incidence of outcome (except for all-cause mortality), frailty status, and number of GP consultations during the lead-in period. CI = confidence interval. Q4 = quartile with highest continuity. Q3 = high intermediate quartile. Q2 = low intermediate quartile.***

Patients in the HQ of the UPC, compared with those in the quartile with least continuity (LQ), displayed reduction in risk of delirium by 34.8% (odds ratio [OR] 0.65, 95% confidence interval [CI] = 0.51 to 0.84, *P*<0.01), reduction in risk of incontinence by 57.9% (OR 0.42, 95% CI = 0.31 to 0.58, *P*<0.01), and reduction in risk of emergency admission to hospital by 9.7% (OR 0.90, 95% CI = 0.82 to 0.99, *P* = 0.03), (Figure 1). Dose–response was observed for the intermediate quartiles (Figure 1). The BB and SECON indices produced similar findings, although for the SECON the association with reduced risk of hospital admissions was not significant (Supplementary Table S4).

Results remained stable after excluding individuals in the lowest and highest quintiles of number of consultations with GPs (Supplementary Table S5), and after restricting analyses to individuals living in the community (Supplementary Table S6). Censoring the first 6 months of follow-up did not significantly alter the association with delirium and incontinence, although emergency admission to hospital became non-significant (Supplementary Table S7).

### Treatment of patients with dementia

Patients in the HQ of the UPC had fewer prescriptions (mean 8.52, SD 4.75) than those in the LQ (mean 9.67, SD 5.31, *P*<0.01). A fully adjusted negative binomial regression model confirmed a dose–response relationship with fewer medications by increasing quartiles of UPC (Table 2). The HQ also had a reduced risk of extreme polypharmacy compared with the LQ (OR 0.83, 95% CI = 0.73 to 0.95, *P*<0.01). The BB and SECON indices produced similar results, although without a dose–response in unadjusted models (Table 2 and Supplementary Table S8).

**Table 2.** Number of drugs and prevalence of potentially inappropriate prescribing by quartiles of continuity of GP care

**Quartile**	**Number of prescriptions**				**Potentially inappropriate prescribing**			
	**Mean (SD)**	***P-*value^a^**	**IRR (95% CI)^b^**	***P-*value^b^**	**Mean (SD)**	***P* value ^a^**	**IRR (95% CI)^b^**	***P-*value^b^**
**Usual Provider of Care Index**								
Lowest quartile	9.67 (5.31)	—	ref	—	2.50 (2.28)	—	ref	—
Low intermediate quartile	9.47 (5.17)	0.20	0.99 (0.96 to 1.01)	0.33	2.47 (2.18)	1.00	1.00 (0.95 to 1.04)	0.89
High intermediate quartile	8.98 (4.93)	<0.01	0.97 (0.94 to 0.99)	0.01	2.29 (2.17)	<0.01	0.96 (0.92 to 1.01)	0.09
High quartile	8.52 (4.75)	<0.01	0.96 (0.93 to 0.98)	<0.01	2.09 (2.06)	<0.01	0.93 (0.88 to 0.97)	<0.01
**Bice–Boxerman Continuity of Care Index**								
Lowest quartile	9.35 (5.23)	—	ref	—	2.40 (2.23)	—	ref	—
Low intermediate quartile	9.29 (5.08)	0.56	0.98 (0.95 to 1.01)	0.11	2.41 (2.22)	0.71	0.99 (0.94 to 1.04)	0.60
High intermediate quartile	9.47 (5.12)	0.36	0.98 (0.96 to 1.01)	0.13	2.44 (2.19)	0.25	0.98 (0.93 to 1.03)	0.41
High quartile	8.56 (4.77)	<0.01	0.96 (0.93 to 0.99)	<0.01	2.09 (2.06)	<0.01	0.93 (0.88 to 0.97)	<0.01
**Sequential Continuity Index**								
Lowest quartile	9.10 (5.10)	—	ref	—	2.30 (2.20)	—	ref	—
Low intermediate quartile	9.70 (5.15)	<0.01	0.98 (0.95 to 1.00)	0.11	2.56 (2.22)	<0.01	0.99 (0.95 to 1.04)	0.80
High intermediate quartile	9.36 (5.22)	0.12	0.98 (0.96 to 1.01)	0.15	2.41 (2.20)	0.04	0.98 (0.93 to 1.02)	0.33
High quartile	8.51 (4.71)	<0.01	0.96 (0.93 to 0.98)	<0.01	2.08 (2.07)	<0.01	0.93 (0.89 to 0.98)	<0.01

a *Two-sided student* t*-test.*

b *IRRs estimated using negative binomial regression model, stratified by quartile of CGPC and adjusted for age, sex, 14 comorbidities, and frailty status. CGPC = continuity of GP care. CI = confidence interval. IRR = incidence rate ratio. ref = reference. SD = standard deviation.*

Patients in the HQ of the UPC had significantly fewer instances of PIP (mean 2.09, SD 2.06) compared with the LQ (mean 2.50, SD 2.28, *P*<0.01). Negative binomial regression models confirmed this reduction in PIP was statistically significant (Table 2). Higher levels of CGPC were not associated with the likelihood of having ≥1 PIP. The BB and SECON indices produced similar results, although without a dose–response in unadjusted models (Table 2 and Supplementary Table S8).

Patients in the HQ of the UPC, compared with the LQ, were 12.3% less likely to be prescribed loop diuretics for treatment of hypertension in patients with urinary incontinence (OR 0.88, 95% CI = 0.78 to 0.99, *P* = 0.03); 25.4% less likely to receive benzodiazepines if at risk of falling (OR 0.75, 95% CI = 0.62 to 0.89, *P*<0.01), 6.7% less likely to receive drugs likely to cause constipation (OR 0.93, 95% CI = 0.89 to 0.98, *P* = 0.01); 15.4% less likely to receive corticosteroids (other than periodic intra-articular injections for mono-articular pain) for osteoarthritis (Table 3). The BB and SECON indices produced similar findings, although the SECON reduction in prescription of loop diuretics to patients with incontinence was not significant (Supplementary Table S9). Also, patients in the HQ of the BB and SECON were more likely to receive benzodiazepines lasting over 1 month (BB OR 1.18, 95% CI = 1.04 to 1.33, *P*<0.01; SECON OR 1.13, 95% CI = 1.00 to 1.28, *P* = 0.05) (Supplementary Table S9).

**Table 3.** Association between prevalence of potentially inappropriate prescribing by quartile of Usual Provider of Care Index

**STOPP criteria V2**	**OR (95% CI)^a^**		
	**Low intermediate quartile**	**High intermediate quartile**	**High quartile**
Loop diuretic for hypertension + urinary incontinence	0.88 (0.64 to 1.20)	0.93 (0.79 to 1.09)	0.88 (0.78 to 0.99)^b^
Benzodiazepines with high risk of falls	0.65 (0.42 to 1.01)	0.57 (0.43 to 0.75)^b^	0.75 (0.62 to 0.89)^b^
Drugs likely to cause constipation	0.90 (0.79 to 1.04)	0.93 (0.87 to 1.00)	0.93 (0.89 to 0.98)^b^
Corticosteroids (other than periodic intra-articular injections for mono-articular pain) for osteoarthritis	0.82 (0.54 to 1.23)	0.72 (0.56 to 0.92)^b^	0.84 (0.72 to 0.99)^b^

a *Compared with lower quartile for Usual Provider of Care Index.*

b P*-value* <*0.05, logistic regression adjusted for age, sex, the diagnosis of 14 chronic conditions, frailty status, and number of GP consultations during the lead-in period. CI = confidence interval. OR = odds ratio.*

## DISCUSSION

### Summary

In this study the continuity in general practice for people with dementia was investigated. Higher levels of CGPC were associated with a reduction in incidence of delirium, incontinence, and emergency hospital admission. For the UPC, this represented a 34.8% reduction in incident delirium, 57.9% reduction in incident incontinence, and a 9.7% reduction in incident emergency admissions to hospital. Comparisons with the least continuity quartiles and intermediate quartiles showed a dose–response relationship suggesting even small increases in CGPC may benefit patients.

AHOs were common in patients with dementia, with 39% experiencing an emergency admission to hospital, 5.2% experiencing delirium, and 3.5% experiencing incontinence during the follow-up period. For delirium, which is underdiagnosed, incidence may be higher.^22^^,^^23^ Patients with dementia have an increased risk for developing delirium, three times the risk of incontinence, and almost 50% more risk of admission to hospital compared with patients without dementia.^23^^–^25

Therefore a reduction in risks through CGPC can be particularly beneficial.

People with dementia have characteristically high levels of comorbidity (92% in this sample),^26^ and Guo *et al* have shown the effect of continuity in reducing drug–drug interaction increases with comorbidity.^27^ Benefits to health may extend beyond prevention of AHOs as higher continuity of care has also been associated with slower progression of comorbid conditions.^28^ Patients with dementia are therefore a key patient group to benefit from higher CGPC.

The results in the current study show higher CGPC leads to better health outcomes, at least in part by reducing inappropriate medication.^6^^,^^29^ Higher CGPC was also associated with fewer drugs deemed potentially inappropriate and with lower medication burden. High medication burden, although not always inappropriate, has been linked to worse health outcomes.^30^ Specifically, patients in the HQ were less likely to be prescribed benzodiazepines if at risk of falling and drugs likely to cause constipation — drug interactions that increase the risk of delirium. This group was also less likely to receive loop diuretics for the treatment of hypertension in individuals with concurrent urinary incontinence, which can exacerbate incontinence symptoms. This reduction in drug prescribing is consistent with the observed reductions in risk of incident delirium and incontinence found in patients in the HQ of CGPC, indicating improved medication management may contribute to the gains in health associated with higher CGPC.^6^^,^^29^^,^^31^

### Strengths and limitations

The large study sample is broadly representative of patients living with dementia in England. CGPC, as well as treatment and AHOs, have been characterised using ‘real-world’ data from general practices, with accurate data on GP-recorded diagnoses and prescriptions. HES data was used to ascertain outcomes during the follow-up. CGPC was calculated using the UPC, BB, and SECON indices, which are established and peer-reviewed algorithms.^16^^,^^17^ In total, 1851 individuals with less than three GP visits were excluded from the study. These are likely comparatively younger and healthier patients and less dependent on healthcare services. The observational nature of this study provides data on statistical associations but cannot indicate causation. This study has, nonetheless, produced robust analyses including adjustment for 14 chronic comorbidities, frailty, and use of health services. Results remained stable after excluding the patients with uncharacteristically low or high consultations and when focusing on patients living in the community to minimise the effect of extreme frailty that was not controlled for by the adjustments.

Study design and sensitivity analyses excluding the first 6 months of follow-up minimise the potential role of reverse-causation driving the findings. Finally, the number of PIP criteria available means associations with CGPC may be affected by false discovery rates and additional studies are required to reproduce these findings.

### Comparison with existing literature

Limited evidence is available on the impact of CGPC in people living with dementia. This study describes novel associations between CGPC and a sizeable reduction in the risk of AHOs delirium and incontinence. Delirium and incontinence are the AHOs with the greatest risk reduction. These may also explain the reduction in hospitalisations. These are important findings for patients with dementia, as delirium often leads to institutionalisation, more admissions to hospital, and death, and incontinence is a humiliating condition that places significant burden on carers.^32^^,^^33^

The findings in the current study are consistent with previous studies asserting that higher continuity of GP care is associated with reduced rates of admission to hospital for patients with dementia,^34^^,^^35^ and for older patients in general.^36^ Unlike in previous non-dementia specific studies, in this study an association with all-cause mortality was not found;^9^^,^^10^ however, this study had a comparatively short 1-year follow-up and patients with dementia have higher mortality rates, which may have affected estimates.^37^ The findings on prescribing are also consistent with those from hospital data that found that continuity of care was associated with a reduction in potentially inappropriate medication.^26^^,^^29^

### Implications for practice and research

Treatment plans are complicated for patients with dementia, who often have multiple diseases.^4^ Patients with dementia can be prescribed PIP from both general practice and hospitals, where 66% of patients are discharged with a PIP.^38^ GPs play a key role in managing medication regimes and the ability to see the same GP rather than, for example, a locum (that is, greater continuity) can contribute to better medication management and fewer PIP.

Continuity in general practices has been falling in recent years;^39^ initiatives focusing on ease of access have had an impact on CGPC along with changes to practice organisation, and the lack of funding for implementation.^40^ The next step is to encourage the provision of more GP continuity. Education on the value of continuity research is needed for undergraduates, postgraduates, and in continuing professional development. Successful implementation, meaning that all patients have the opportunity for continuity, requires additional research on implementation strategies, such as personal lists and measurement of continuity, and support by the Department of Health. A personal list is a patient management approach for GP practices (with multiple GPs, where patients are assigned to a specific GP and then encouraged to consult with them consistently, especially in situations when major decisions about disease/case management are required).^41^

Continuity of midwifery care is policy in the NHS Long Term Plan; similar policy is needed for general practice.^42^^,^^43^

Continuity of care is recognised as an important step for improving dementia care.^11^ Patients with dementia are particularly vulnerable to the pressures currently placed on general practices because of high workloads, limited funding, and recruitment difficulties,^44^^,^^45^ and in such circumstances these patients can receive lower standards of care and often lower CGPC.^46^ Although significant work remains on the implementation of CGPC in practices, prioritising patients with dementia in the meantime, by allowing them to consistently access their named GP, can help prevent AHOs and contribute to better medication management, and by extension lead to better health and quality of life.

## Data

For access to study protocol and programming code contact the corresponding author, João Delgado. The raw data are available from Clinical Practice Research Datalink.
